# Targeting LINC00707 by vitamin D3 attenuates nitrogen mustard-caused dermal toxicity through inhibiting ferroptosis

**DOI:** 10.1016/j.redox.2025.103628

**Published:** 2025-04-10

**Authors:** Xunhu Dong, Ying He, Xiaofeng Hu, Jie Wu, Feng Ye, Xiaogang Wang, Yuanpeng Zhao, Guorong Dan, Jiqing Zhao, He Tang, Xiaolu Lu, Yan Sai, Zhongmin Zou, Mingliang Chen

**Affiliations:** a Institute of Toxicology, School of Military Preventive Medicine, Army Medical University, Chongqing, 400038, China; b Institute of Pathology and Southwest Cancer Centre, Southwest Hospital, Army Medical University, Chongqing, 400038, China; c Department of Ultrasound, Xinqiao Hospital, Army Medical University, Chongqing, 400037, China; d Chinese PLA Center for Disease Control and Prevention, Beijing, China; e Department of Tropical Medicine, School of Military Preventive Medicine, Army Medical University, Chongqing, 400038, China; f State Key Laboratory of Trauma and Chemical Poisoning, China

**Keywords:** Vitamin D3, Nitrogen mustard, LINC00707, Nrf2, Ferroptosis

## Abstract

Nitrogen mustard (NM) causes severe skin injury that is lack of effective and targeted therapies. Vitamin D3 (VD3) emerges as a promising treatment option for NM-caused dermal toxicity; however, the underlying mechanisms are currently unclear. Herein, we identified that NM markedly promoted ferroptosis by measurement of decreased cell viability, glutathione, glutathione peroxidase 4 and solute carrier family 7 member 11 levels, and increased ROS, lipid ROS, iron/Fe^2+^ and malondialdehyde contents in vitro and in vivo. Ferrostin-1 (Fer-1, a ferroptosis inhibitor) attenuated NM-caused cell death in keratinocytes. Meanwhile, NM significantly inhibited phosphorylation of AKT1 and glycogen synthase kinase 3β (GSK3β) and nuclear factor erythroid 2-related factor 2 (Nrf2) nuclear translocation, and increased LINC00707 expression. Furthermore, NM-induced ferroptosis in keratinocytes was abolished by treatment with agonists of Nrf2 (tBHQ) and AKT1 (SC79), the inhibitor of GSK3β (AR-A014418), Nrf2 overexpression or LINC00707 knockdown. Mechanistically, LINC00707 directly bound with the protein kinase domain of AKT1 and suppressed its phosphorylation and activated GSK3β thereby inactivating Nrf2, subsequently inducing ferroptosis and cell death in NM-treated keratinocytes. Moreover, VD3 notably suppressed LINC00707 expression, activated AKT1 and inactivated GSK3β, increased Nrf2 nuclear translocation and inhibited ferroptosis and cytotoxicity induced by NM in vitro and in vivo. The protective effects of VD3 against NM-caused dermal toxicity were blocked by erastin (a ferroptosis inducer), *Nrf2* siRNA, LINC00707 overexpression and were enhanced by LINC00707 knockdown and Fer-1 in vitro and in vivo. In conclusion, VD3 ameliorated NM-caused dermal toxicity by inhibiting ferroptosis, which was partially mediated through the LINC00707-AKT1-GSK3β-Nrf2 signaling pathway.

## Highlights

• Ferroptosis plays a critical role in NM-induced dermal toxicity.
• NM induces ferroptosis through the LINC00707-AKT1-GSK3β-Nrf2 signaling pathway.
• Targeting LINC00707 by VD3 alleviates NM-caused skin injury via inhibiting ferroptosis.

## Graphical abstract

**Figure:**
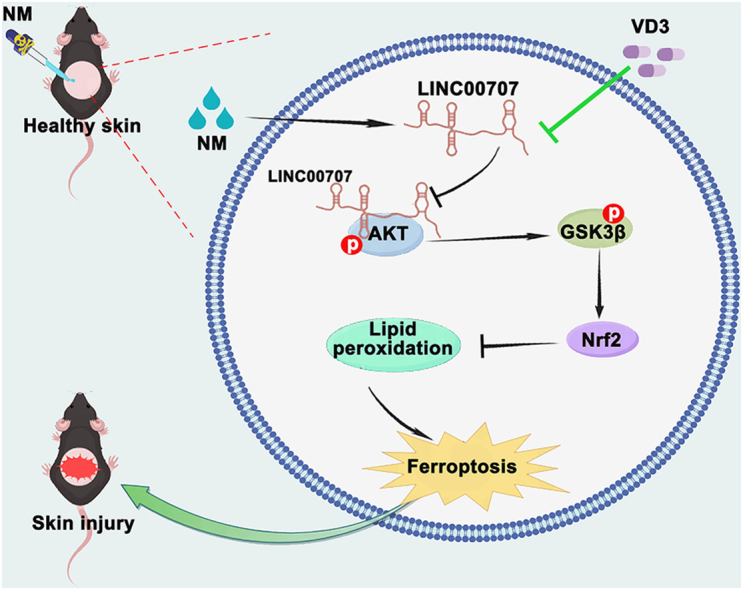

## 1 Introduction

Nitrogen mustard (NM) is an extremely toxic blister agent that has been demonstrated to elicit detrimental effects across a variety of organ systems. Among which, the skin has been considered as the primary and most vulnerable target for NM [1,2]. After exposure, NM swiftly permeates into the epidermis, thereby triggering a pronounced oxidative stress and inflammatory response, cell death, alongside with delayed excruciating vesication and refractory ulcers [1,2]. Until now, effective therapies for NM-induced skin injury are still lack, for the poor understanding of the underlying mechanism(s).

Ferroptosis is an iron-dependent programmed cell death triggered by cellular response to impaired glutathione (GSH) metabolism, iron accumulation and oxidation-reduction imbalance, which is genetically, biochemically, and morphologically distinct from other classical types of cell death such as apoptosis, pyroptosis, and necroptosis [3,4]. Recently, it has been demonstrated that ferroptosis is involved in the pathogenesis of diverse skin disorders and injuries [5]. Researchers have found that ferroptosis activation contributes to the formation of skin lesions in psoriasis vulgaris [6] and inhibition of keratinocyte ferroptosis suppresses psoriatic inflammation [7]. Vats et al. reported that ultraviolet B radiation (UVB) induces ferroptosis thereby initiating skin inflammation [8]. Meanwhile, Feng and co-workers confirmed that nicotinamide mononucleotide (NMN) inhibits ferroptosis in UV irradiation-induced skin injury [9]. These results suggest that ferroptosis is plausibly linked to skin damages caused by physical or chemical exposure. In 2024, Assylbek et al. identified that NM treatment of corneal cells results in 228 differentially and exclusively expressed proteins primarily associated with ferroptosis [10], indicating a possible role of ferroptosis in NM-mediated cytotoxicity. However, there is a lack of information pertaining to the involvement of ferroptosis in NM-induced dermal toxicity and the mechanisms underpinning it.

The nuclear factor erythroid 2-related factor 2 (Nrf2), a crucial modulator in cellular oxidative stress response, has been identified as the primary negative regulator of ferroptosis by orchestrating the expression of pivotal proteins for iron homeostasis and enzymes indispensable for GSH synthesis, nicotinamide adenine dinucleotide phosphate generation, and lipid peroxidation [11]. Among which, the usually used biomarkers of ferroptosis: cystine-glutamate antiporter (SLC7A11, a critical regulator of GSH synthesis) and glutathione peroxidase 4 (GPX4, a crucial enzyme for lipid peroxidation), have been found to be the most important transcriptional targets of Nrf2 [12,13]. Reportedly, the protein kinase B (PKB/AKT)-glycogen synthase kinase-3β (GSK3β) pathway is essential for the regulation of Nrf2 activity by directly phosphorylating the Neh 6 domain of Nrf2, which has been proposed as a core signaling pathway for ferroptosis regulation [[14], [15], [16]]. Moreover, the latest studies unveiled that long noncoding RNAs (LncRNAs, a class of RNA transcripts longer than 200 nucleotides without protein-coding properties) participate in diverse diseases through regulating Nrf2-mediated ferroptosis [[17], [18], [19]]. More recently, Yo et al. found that UV irradiation can cause significant changes in LncRNA expression profiles in keratinocytes, with UVA causing 660 LncRNA expression changes and UVB causing 3559 LncRNA expression changes [20]. Meanwhile, LncRNA AGAP2-AS1 upregulates AKT activity thereby improving the proliferation of keratinocytes, ultimately promoting psoriasis pathogenesis [21]; downregulation of LINC00672 shortens the time to convert human skin fibroblasts into keratinocyte-like cells from 110 d to 14 d by inhibiting AKT3, providing a new avenue for skin wound healing [22]. These findings indicate that LncRNAs might emerge as an upstream molecular target against skin injuries by regulating Nrf2-mediated ferroptosis via the AKT-GSK3β pathway. However, the role of LncRNAs in NM-induced dermal toxicity is not clear.

Vitamin D3 (VD3) is a steroid hormone synthesized in skin that is able to attenuate NM-induced skin injury; however, limited information is available regarding the underlying mechanisms. Our prior research supported that VD3 ameliorates NM-induced skin damage through reducing reactive oxygen species (ROS) generation thereby inhibiting the NLRP3 inflammasome-mediated inflammatory response [23]. Moreover, VD3 attenuates osteoblastic ferroptosis by stimulating the Nrf2/GPX4 pathway in age-related osteoporosis and improves cognitive impairment by alleviating ferroptosis via the Nrf2 signaling pathway in aging mice [24,25]. It has also been found that VD3 upregulates the Nrf2 signaling pathway to suppress ferroptosis thereby protecting against diabetic nephropathy and neonatal hypoxic-ischemic encephalopathy [26,27]. These published data imply the involvement of Nrf2-mediated ferroptosis in VD3's favorable outcomes. Nevertheless, the potential significance of ferroptosis in mediating VD3's protective action against NM-caused dermal toxicity necessitates further elaboration.

For the first time, our current findings validated that VD3 efficiently safeguarded keratinocytes from NM-elicited dermal toxicity by suppressing ferroptosis, which was partially mediated by the LINC00707-AKT1-GSK3β-Nrf2 signaling pathway. These findings offer a novel molecular basis for VD3, which could potentially be harnessed in the treatment of NM-induced dermal toxicity.

## 2 Materials and methods

### 2.1 Cell culture and treatments

HaCaT cells were purchased from Chinese Academy of Sciences Shanghai Cell Bank (cs0014, Shanghai, China) and cultured in Roswell park memorial institute (RPMI)-1640 supplemented with 10 % fetal bovine serum in a humidified atmosphere containing 5 % CO_2_ at 37 °C. The medium was changed at 2-day intervals and cells re-plated at 80–90 % confluence. During the logarithmic growth phase, cells were treated with NM (20 μM) for 24 h. Where indicated, cells were treated with erastin (100 μM) [28], ferrostatin-1 (Fer-1, 10 μM) [8], tBHQ (20 μM) [29], ML385 (10 μM) [30], SC79 (10 μM) [31], A-674563 (100 nM) [32], or AR-A014418 (AR, 10 μM) [33] for 1 h, following incubation with NM (20 μM) without washing for another 24 h in the presence or absence of VD3 (calcitriol, 10 nM) [23]. All the inhibitors or activators were dissolved in dimethyl sulfoxide (DMSO) and subsequently diluted to the required working concentration using full RPMI-1640 medium. And the control group was treated with 0.1 % DMSO.

### 2.2 Animals and treatments

Eight-week-old male C57BL/6J mice weighing 22–24 g were acquired from the Animal Center of Army Medical University (Chongqing, China) and maintained on a standard laboratory diet. Approval for all animal experiments was granted by the Animal Care and Use Committee of the Army Medical University (AMUWEC20225718, Chongqing, China).

To create skin wounds, mice were anesthetized through an intraperitoneal injection of pentobarbital sodium anesthetic at a dose of 50 mg/kg. The dorsal fur was then removed using clippers and depilating cream. After 48 h, dorsal skin of mice (n = 6 per group) was exposed to 3.2 mg NM in 200 μL acetone while control mice received 200 μL acetone only, as described earlier [34]. VD3 was reconstituted in DMSO and diluted in mineral oil for intraperitoneal injection at a concentration of 50 ng VD3 (cholecalciferol)/100 μL per mouse [23] combined with or without erastin (25 mg/kg) [35], at 1 h after NM exposure. Meanwhile, mice dorsal skin were treated with 200 μL total volume of Fer-1 (10 μM, diluted in 70 % ethanol, twice within 24 h) [8] at 1h after NM exposure, combined with VD3 (50 ng/100 μL per mouse, i.p.) as previously reported [8]. Adenovirus-associated virus carrying the keratinocyte-specific promoter Krt14 with LINC00707 (AAV-707OE, Genechem, Shanghai, China) and AAV-Control (AAV-CT, Genechem, Shanghai, China) were delivered subcutaneously via a 35-gauge needle (Hamilton, Martinsried, Germany) around the specific region of dorsal skin. A total of five injections, each containing approximately 10 μL at a concentration of 1.8 × 10^13^ viral genomes, were administered as described before [36]. Three weeks after injection, mice were treated with NM and VD3 as described above. At 4 h after NM exposure, exposed regions of skin were gently wiped with sodium hypochlorite (0.8 %) and saline for decontamination. Images of wounds were obtained with a digital camera on days 1, 3, and 7. Mice were sacrificed at 7 d following NM exposure. Dorsal skin tissue was collected and fixed in 10 % formalin for histopathological analysis or snap-frozen in liquid nitrogen for Western blot or other analysis.

### 2.3 Statistical analyses

Quantitative data are presented as the means ± standard deviation (SD) of four independent experiments. The statistical analysis was performed utilizing the *t*-test and one-way analysis of variance within SPSS 23.0 statistical software (SPSS Inc., Chicago, IL, USA). Statistical significance was assigned to a *P*-value of less than 0.05 and the Tukey-Kramer post-hoc test was applied if *P* < 0.05.

Full descriptions of additional materials and methods are given in the Supporting Information.

## 3 Results

### 3.1 NM caused cytotoxicity by inducing ferroptosis in keratinocytes

**Fig. 1:**
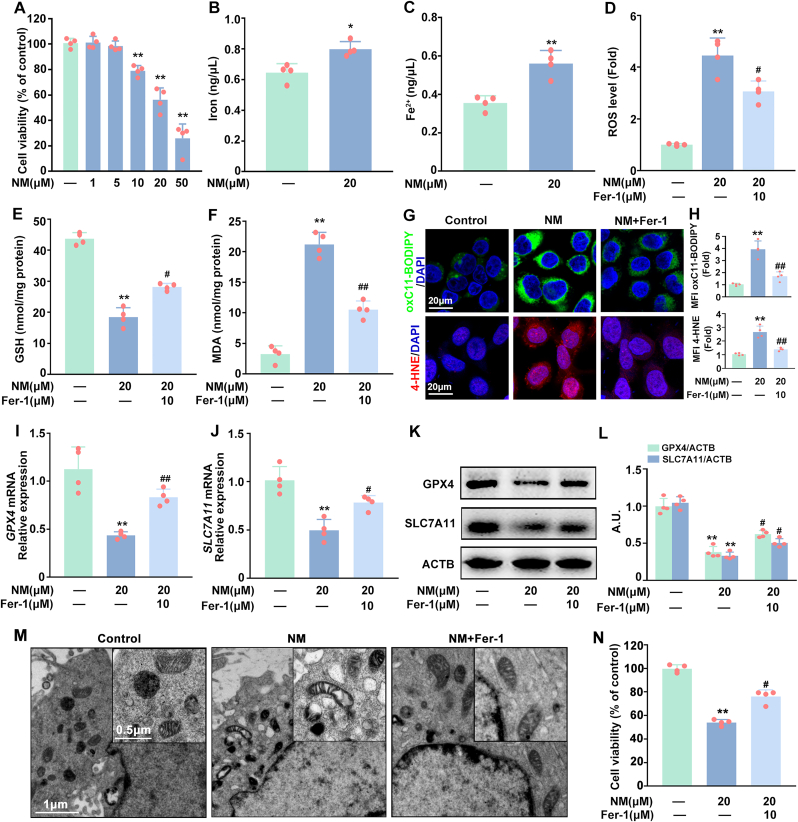
**NM caused cytotoxicity by inducing ferroptosis in keratinocytes.** (**A**) Cells were incubated with different concentrations of NM (0, 1, 5, 10, 20, 50 μM) for 24 h and cell viability were determined by a cell counting kit-8 (CCK-8) detection kit. Cells were treated with NM (20 μM) for 24 h **(B)** Total iron and **(C)** Ferrous iron (Fe^2+^) were measured via a specific iron assay kit. Cells were pretreated with Fer-1 (10 μM) for 1 h and exposed to NM (20 μM) for a further 24 h. **(D)** Detection of total ROS levels using DCFH-DA. Measurement of **(E)** GSH and **(F)** MDA levels by specific assay kits. **(G)** Representative fluorescence images of HaCaT cells stained with C11-BODIPY or 4-HNE. **(H)** Relative mean fluorescence intensity (MFI) of oxidized (ox) C11-BODIPY or 4-HNE. Relative expression of **(I)***GPX4* and **(J)***SLC7A1*1 mRNA determined by qRT-PCR. (**K**) Western blot analysis of GPX4 and SLC7A11 expression. **(L)** The bar graphs show the quantification of the indicated proteins. **(M)** Mitochondrial morphological changes were detected by transmission electron microscopy. **(N)** Cell viability was measured by a CCK-8 kit. Values are presented as means ± SD (n = 4); ∗*p* < 0.05, ∗∗*p* < 0.01 versus vehicle-treated control group; ^#^*p* < 0.05, ^##^*p* < 0.01 versus NM-treated group; A.U. arbitrary units.

To determine the role of ferroptosis in NM-mediated cytotoxicity in keratinocytes, HaCaT cells were exposed with a series concentration of NM (0, 1, 5, 10, 20, and 50 μM) for 24 h. In line with our previous findings [37], NM significantly inhibited HaCaT cells’ viability in a dose-dependent manner and when the concentration of NM reached 20 μM, the cell viability decreased to approximately 60 % (Fig. 1A). Consequently, 20 μM NM was employed in subsequent experiments to elucidate the role of ferroptosis and the underlying mechanism in NM-induced dermal toxicity. As shown in Fig. 1B–F, NM markedly increased the contents of intracellular iron/Fe^2+^, ROS, malondialdehyde (MDA), and decreased GSH levels in keratinocytes. Meanwhile, NM induced the generation of lipid ROS (determined by BODIPY™ 581/591 C11 (C11-BODIPY), a special intracellular lipid ROS probe) and 4 Hydroxynonenal (4-HNE, a lipid peroxidation product) in keratinocytes (Fig. 1G and H). Likewise, the levels of GPX4 and SLC7A11 mRNA and protein (the widely used ferroptosis markers [38]) were also notably inhibited by NM in keratinocytes (Fig. 1I-L). Moreover, the changes of mitochondrial morphology (another typical characteristic of ferroptosis [38]) was investigated by transmission electron microscope to further confirm NM-induced ferroptosis in keratinocytes. As depicted in Fig. 1M, NM caused atrophied mitochondria, compressed membrane density, deceased cristae and ruptured outer membranes in keratinocytes. Additionally, the above-mentioned effects of NM on ROS, MDA, lipid ROS, 4-HNE, GPX4, SLC7A11 and mitochondrial morphology were abolished by Fer-1 (a specific ferroptosis inhibitor, 10 μM), thereby significantly attenuating NM caused inhibition of cell viability of keratinocytes (Fig. 1D-N). These collective results indicated that NM induced ferroptosis that contributed to NM-caused cytotoxicity in keratinocytes.

### 3.2 NM induced ferroptosis through inhibiting Nrf2 in keratinocytes

**Fig. 2:**
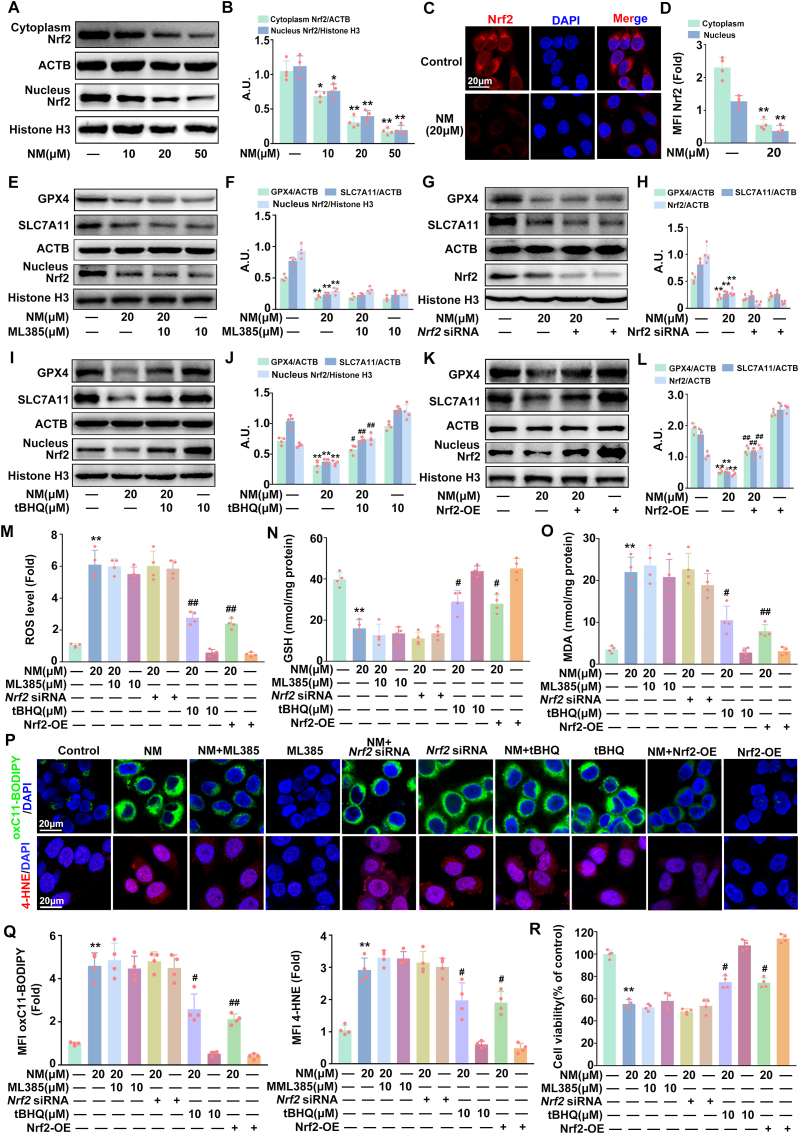
**NM induced ferroptosis through inhibiting Nrf2 in keratinocytes.** Cells were treated with various concentrations of NM (0, 10, 20 and 50 μM) for 24 h. **(A)** The expression of Nrf2 protein in cytoplasm and nucleus was detected by Western blot. **(B)** The bar graphs show the quantification of the indicated proteins. **(C)** Immunofluorescence staining analysis of Nrf2 in the nucleus and cytoplasm, nuclei were counterstained with DAPI (blue). **(D)** MFI of Nrf2 relative to (C). Cells were pretreated with ML385 (10 μM) or tBHQ (10 μM) for 1 h, *Nrf2* siRNA or lentivirus encoding Nrf2 as described in the Materials and methods, followed by exposure to NM (20 μM) for an additional 24 h. **(E), (G)**, **(I)** and **(K)** Western blot analysis was used to detect the protein levels of GPX4, SLC7A11, Nrf2, ACTB, nuclear Nrf2 and Histone H3. **(F)**, **(H), (J)** and **(L)** The bar graphs show the quantification of the indicated proteins. **(M)** Total ROS levels was detected by DCFH-DA. Measurement of **(N)** GSH and **(O)** MDA with specific assay kits. **(P)** Representative images of oxC11-BODIPY or 4-HNE detected with a ZEISS LSM800 confocal laser scanning microscope. **(Q)** Relative MFI of oxC11-BODIPY or 4-HNE. **(R)** Cell viability was detected by a CCK-8 detection kit. Values are expressed as means ± SD (n = 4); ∗*p* < 0.05, ∗∗*p* < 0.01 versus vehicle-treated control group; ^#^*p* < 0.05, ^##^*p* < 0.01 versus NM-treated group; A.U. arbitrary units.

Nrf2 has been considered as a key regulator of ferroptosis [13]. Therefore, the involvement of Nrf2 in NM-induced ferroptosis was intensively investigated. As shown in Fig. 2A–D, the expression of Nrf2 was dose-dependently decreased both in the cytoplasm and nuclei in NM-treated keratinocytes, indicating the inactivation of Nrf2. Moreover, NM failed to further decrease GPX4 and SLC7A11 expression, GSH levels and increase ROS and MDA, lipid ROS, and 4-HNE generation as well as inhibiting cell viability in the presence of ML385 (a selected Nrf2 inhibitor, 10 μM) or *Nrf2* small interference RNA (siRNA) in keratinocytes (Fig. 2E–H, M-R). However, these effects were significantly abolished by tert-Butylhydroquinone (tBHQ, a widely used Nrf2 agonist, 20 μM) or Nrf2 overexpression (Fig. 2I–R). Our data validated the requirement of Nrf2 for NM-induced ferroptosis and cytotoxicity in keratinocytes.

### 3.3 NM inhibited Nrf2 via the AKT1-GSK3β pathway in keratinocytes

**Fig. 3:**
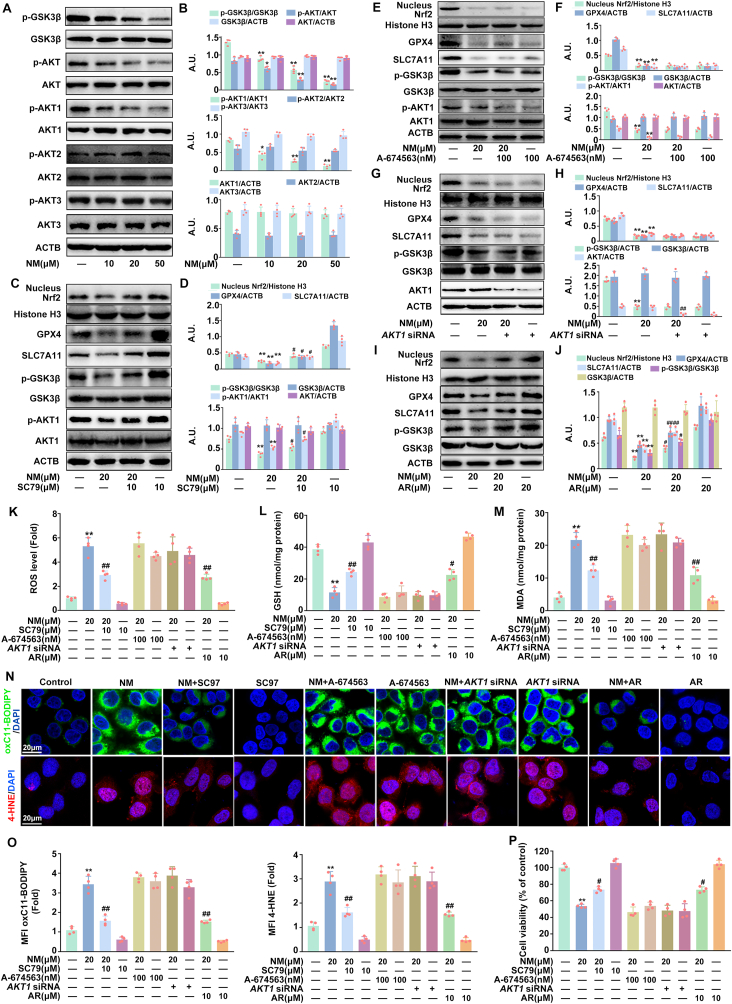
**NM inhibited Nrf2 via the AKT1-GSK3β pathway in keratinocytes.** Cells were treated with various concentrations of NM (0,10, 20, and 50 μM) for 24 h. **(A)** Total cell lysates were immunoblotted with anti-*p*-GSK3β, anti-GSK3β, anti-pAKT, anti-AKT, anti-*p*-AKT1, anti-AKT1, anti-*p*-AKT2, anti-AKT2, anti-*p*-AKT3, anti-AKT3 or anti-ACTB antibodies. **(B)** The bar graphs show the quantification of the indicated proteins. Cells were pretreated with SC79 (10 μM), A-674563 (100 nM), AR (10 μM) for 1 h or *AKT1* siRNA as described in the Materials and methods, and incubated with NM (20 μM) for a further 24 h. **(C)**, **(E), (G)** and **(I)** Western blot analysis of nuclear Nrf2, Histone H3, *p*-GSK3β, GSK3β, *p*-AKT1, AKT1, GPX4, SLC7A11 and ACTB expression. **(D), (F), (H)** and **(J)** The bar graphs show the quantification of the indicated proteins. **(K)** Total ROS levels was detected by DCFH-DA. Measurement of **(L)** GSH and **(M)** MDA with specific assay kits. **(N)** Representative images of oxC11-BODIPY or 4-HNE detected with a ZEISS LSM800 confocal laser scanning microscope. **(O)** Relative MFI of oxC11-BODIPY or 4-HNE. **(P)** Cell viability was detected by a CCK-8 detection kit. Values are expressed as means ± SD (n = 4); ∗*p* < 0.05, ∗∗*p* < 0.01 versus vehicle-treated control group; ^#^*p* < 0.05, ^##^*p* < 0.01 versus NM-treated group; A.U. arbitrary units.

A growing body of evidence indicates that the AKT-GSK3β pathway plays an important role in the regulation of Nrf2-mediated ferroptosis [15,39]. Thus, the effects of NM on AKT and GSK3β activity were determined. The AKT serine/threonine kinase family contains three isoforms (AKT1-3) that have distinct functions. Herein, we found that NM dose-dependently inhibited the expression of *p*-AKT, *p*-AKT1 with a slight and significant decrease of *p*-AKT3, but had no significant effect on *p*-AKT2, indicating the inhibition of AKT especially AKT1 by NM. Likewise, NM decreased *p*-GSK3β expression, suggesting the activation of GSK3β (Fig. 3A and B).

Moreover, SC79 (an activator of AKT1), A-674563 (a selective inhibitor of AKT1), *AKT1* siRNA, or AR (a specific inhibitor of GSK3β) were taken to further clarify the role of AKT1-GSK3β pathway in NM-induced inactivation of Nrf2 and ferroptosis. As shown in Fig. 3C–D and K–P, SC97 attenuated NM-caused decrease of *p*-AKT, *p*-GSK3β, Nrf2 nuclear translocation, GPX4 and SLC7A11 expression, and GSH depletion, increase of ROS, MDA, lipid ROS, 4-HNE contents and inhibition of cell viability in keratinocytes. Meanwhile, NM failed to further inhibit *p*-GSK3β expression, Nrf2 nuclear translocation, and induce forroptosis, inhibition of cell viability in A-674563 or *AKT1* siRNA-treated keratinocytes (Fig. 3E–H and K–P). Additionally, AR significantly reversed NM-caused decrease of *p*-GSK3β expression, Nrf2 inactivation, ferroptosis and cell death in keratinocytes (Fig. 3I-P). These results suggested that the AKT1-GSK3β pathway was required for Nrf2 inactivation and subsequent NM-induced ferroptosis in keratinocytes.

### 3.4 NM inactivated AKT1 by upregulating LINC00707 in kerotinocytes

**Fig. 4:**
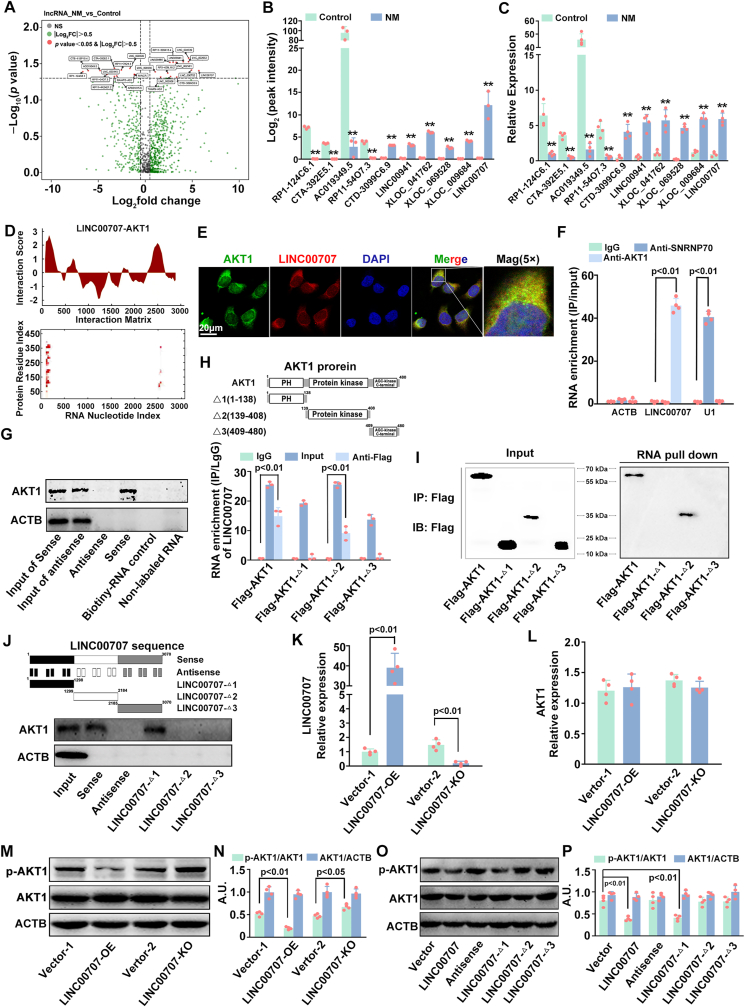
**LINC00707 inhibited AKT1 phosphoralation by interacting with the protein kinase domain. (A)** The different expression of LncRNAs in NM-treated keratinocytes. Red dots mark top regulated LncRNAs (p value < 0.05, **丨** log_2_FC **丨** >0.5). **(B)** Different expression of 10 LncRNAs in NM-stimulated keratinocytes. **(C)** Different expression of LncRNAs (p value < 0.05, **丨** log_2_FC **丨** > 5.0) in NM-stimulated keratinocytes detected by qRT-PCR. **(D)** The catRAPID analysis characterized the binding interaction between LINC00707 and the AKT1 protein. **(E)** A fluorescent in situ hybridization kit was applied to detect the colocalization of LINC00707 with AKT1 protein and was visualized by a ZEISS LSM800 confocal laser scanning microscope. **(F)** The RIP analysis performed on HaCaT cells revealed a significant enrichment of LINC00707 in the immunoprecipitation mixture when AKT1 and IgG antibodies were used. ACTB functioned as the negative control, whereas U1 acted as the positive control. **(G)** Western blot identified that AKT1 protein in the complex coprecipitated pulled down by LINC00707. (H) RIP followed qRT-PCR confirmed the enrichment of LINC00707 by flag-tagged AKT1 (WT and three set of domain truncation mutants) in HaCaT cells. **(I)** Immunoblot detection of flag-tagged AKT1 (WT and three sets of domain truncation mutants) precipitated by transcribed biotinylated-LINC00707 in vitro. **(J)** Immunoblot detection of AKT1, which were pulled down by transcribed biotinylated RNAs corresponding to different fragments of LINC00707 in vitro. The expression levels of (K) LINC00707 and **(L)** AKT1 in HaCaT cells that were transfected with either an overexpression lentivirus or a knockdown lentivirus were detected by qRT-PCR. **(M)** The expression of *p*-AKT1 and AKT1 were detected by Western blot in HaCaT cells transfected with LINC00707 overexpression lentivirus or knockdown lentivirus. **(N)** The bar graphs show the quantification of the indicated proteins. **(O)** The expression of *p*-AKT1 and AKT1 proteins were measured by Western blot in HaCaT cells transfected with exogenous LINC00707 or each of the LINC00707 mutants. **(P)** The bar graphs show the quantification of the indicated proteins. Values are expressed as means ± SD (n = 3/4); ∗∗*p* < 0.01 versus vehicle-treated control group; A.U. arbitrary units.

In view of the finding that LncRNAs play important roles in the regulation of physicochemical stimuli-caused skin injury and AKT activity. Therefore, the involvement of LncRNAs in NM-induced inactivation of AKT1 was also investigated. Via LncRNA sequencing assay, we found that NM led to a total of 13 downregulated LncRNAs and 18 upregulated LncRNAs, among which LINC00707 was the most highly expressed one (fold change >10.0 and p < 0.05) in NM-treated keratinocytes (Fig. 4A and B). To confirm the data acquired by microarray, a subset of differently expressed LncRNAs (fold change >5.0 and p < 0.05) were detected by qRT-PCR analysis. It was also found that LINC00707 expression was the highest, which was in line with the microarray data (Fig. 4C). Previous researches have reported that LINC00707 can regulate variable signaling pathways through directly binding with targeted proteins [40]. As expected, we found that LINC00707 had a remarkable performance with a high protein-RNA interaction propensity to AKT1 by catRAPID (Fig. 4D). Meanwhile, the colocalization of AKT1 with LINC00707 was mainly observed in the cytoplasm of keratinocytes (Fig. 4E). RNA immunoprecipitation (RIP) assay revealed that there was a substantial enrichment of LINC00707 in the immunoprecipitation complex induced by a AKT1 antibody compared to the negative control, with U1 of SNRNP70 as a positive control (Fig. 4F). And AKT1 was detected in the compounds pulled down by biotin-labeled LINC00707 sense (Fig. 4G). These data indicated that LINC00707 can interact with AKT1.

To further characterize the exact domain of AKT1 for binding with LINC00707, three sets of AKT1 protein domain-deletion mutants were taken to perform RIP assays. As shown in Fig. 4H, AKT1 protein domain-deletion mutants Δ2 (139–408 amino acid region, known as the protein kinase domain), but not Δ1 (1–138 amino acid region) or Δ3 (409–480 amino acid region), had the similar ability to bind with LINC00707 as the full-length AKT1 did. Coincidentally, AKT1 protein domain-deletion mutants Δ2 was detected in the full-length LINC00707 pull-down compounds (Fig. 4I). The regions of LINC00707 responsible for its binding with AKT1 were also determined by constructing a series truncations of LINC00707 with RNA pull-down assays. Only truncation Δ1 (1299–2184 nt) of LINC00707 was found to bind with AKT1 (Fig. 4J). Moreover, keratinocytes were transfected with LINC00707 overexpression (LINC00707-OE) or knockout (LINC00707-KO) lentivirus to investigate the regulatory effects of LINC00707 on AKT1 activity. As indicated in Fig. 4K-N, LINC00707-OE significantly increased the expression of LINC00707 thereby decreasing *p*-AKT1 expression, which was reversed by LINC00707-KO; both LINC00707-OE and LINC00707-KO had no significant effect on the expression of AKT1 mRNA and protein. In addition, we found that LINC00707 Δ1 overexpression decreased *p*-AKT1 expression that was similar to those of LINC00707-OE-tansfected group; there was no significant difference of *p*-AKT1 expression among LINC00707 Δ2, Δ3, antisense overexpression and the control group (Fig. 4O and P). These results suggested that LINC00707 interacted with AKT1 protein, and 1299–2184 nt of LINC00707 had strong affinity for the AKT1 protein at the 139–408 amino acid region, which resulted in the inactivation of AKT1.

**Fig. 5:**
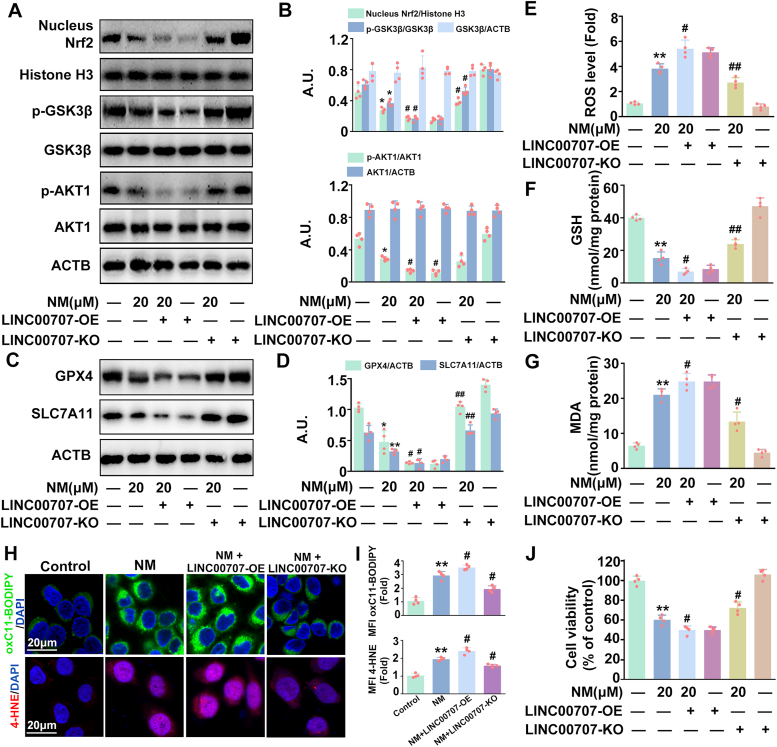
**NM inactivated AKT1 by upregulating LINC00707 in kerotinocytes.** Cells were transfected with LINC00707 overexpression lentivirus or knockout lentivirus followed by exposure to NM for an additional 24 h. **(A)** The proteins levels of nuclear Nrf2, Histone H3, *p*-GSK3β, GSK3β, *p*-AKT1, AKT1 and ACTB were detected by Western blot. **(B)** The bar graphs show the quantification of the indicated proteins. **(C)** Western blot analysis of GPX4 and SLC7A11 expression. **(D)** The bar graphs show the quantification of the indicated proteins. **(E)** Detection of ROS levels using DCFH-DA. **(F)** GSH and **(G)** MDA contents were detected with specific assay kits following the manufacturer's instructions. **(H)** Representative images of oxC11-BODIPY or 4-HNE detected with a ZEISS LSM800 confocal laser scanning microscope. **(I)** Relative MFI of C11-BODIPY or 4-HNE. **(J)** Cell viability was detected by a CCK-8 detection kit. Values are expressed as means ± SD (n = 4); ∗*p* < 0.05, ∗∗*p* < 0.01 versus vehicle-treated control group; ^#^*p* < 0.05, ^##^*p* < 0.01 versus NM-treated group; A.U. arbitrary units.

Additionally, LINC0070-OE transfection enhanced the NM-stimulated decrease of *p*-AKT1, *p*-GSK3β expression and Nrf2 nuclear translocation, thereby, enhancing NM-caused ferroptosis and cell death in keratinocytes; meanwhile, these effects were markedly abolished by LINC0070-KO transfection (Fig. 5A–J). These results, combined with the above data, collaborativelyindicated that LINC00707 was required for AKT1 inactivation and the subsequent activation of the GSK3β-Nrf2-ferroptosis signaling pathway induced by NM in keratinocytes.

### 3.5 VD3 protected keratinocytes against NM-induced cytotoxicity via inhibiting ferroptosis

**Fig. 6:**
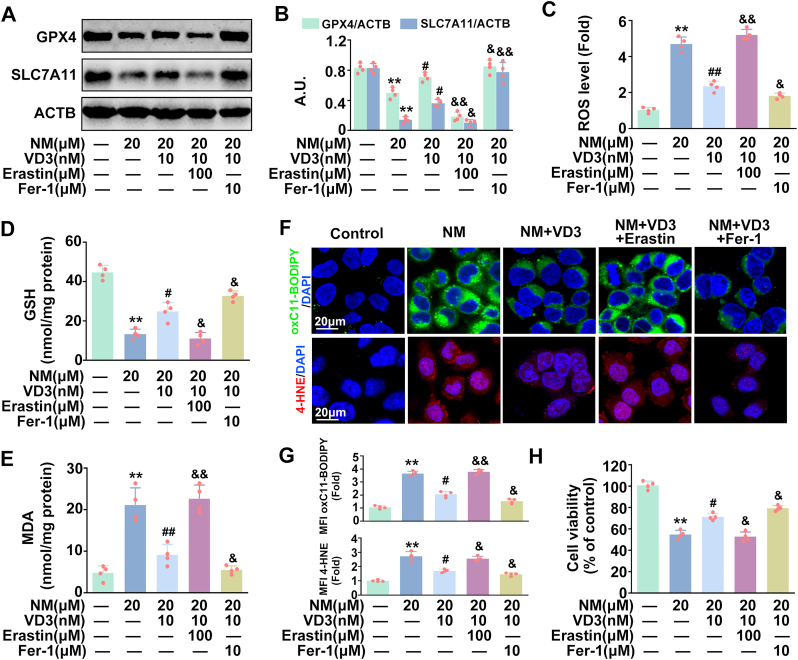
**VD3 protected keratinocytes against NM-induced cytotoxicity via inhibiting ferroptosis.** Cells were pretreated with VD3 (10 nM) for 1 h in the presence or absence of Fer-1 (10 μM) or erastin (100 μM), followed by treatment with NM (20 μM) for another 24 h. **(A)** Western blot analysis of GPX4 and SLC7A11 proteins levels. **(B)** The bar graphs show the quantification of the indicated proteins. **(C)** Detection of ROS levels using DCFH-DA. **(D)** Assay of GSH content with a specific kit following the manufacturer's instructions. **(E)** MDA levels were detected by a lipid peroxidation assay kit. **(F)** Representative images of oxC11-BODIPY or 4-HNE detected with a ZEISS LSM800 confocal laser scanning microscope. **(G)** Relative MFI of oxC11-BODIPY or 4-HNE. **(H)** Cell viability was detected by a CCK-8 detection kit. Values are expressed as means ± SD (n = 4); ∗∗*P* < 0.01 vs. the vehicle-treated control group; ^#^*P* < 0.05, ^##^*P* < 0.01 vs. NM-treated group. ^&^*P* < 0.05, ^&&^*P* < 0.01 vs. NM and VD3 co-treated group.

VD3 has recently been considered as a good candidate for the prevention and treatment of NM-mediated skin damage [23], however limited information is available regarding the underlying mechanisms. According to our above findings, we intended to identify the involvement of ferroptosis in VD3's beneficial effect on NM-induced dermal toxicity. As expected, VD3 significantly attenuated NM-caused decrease of GPX4 and SLC7A11 expression, GSH depletion, the increase of ROS, MDA, lipid ROS and 4-HNE generation and cell death in keratinocytes; however, these effects were notably abolished by erastin and enhanced by Fer-1 (Fig. 6A–H). These results clearly indicated that VD3 attenuated NM-triggered cytotoxicity in a ferroptosis dependent manner.

### 3.6 VD3 inhibited ferroptosis via the LINC00707-AKT1-GSK3β-Nrf2 signaling pathway in keratinocytes

**Fig. 7:**
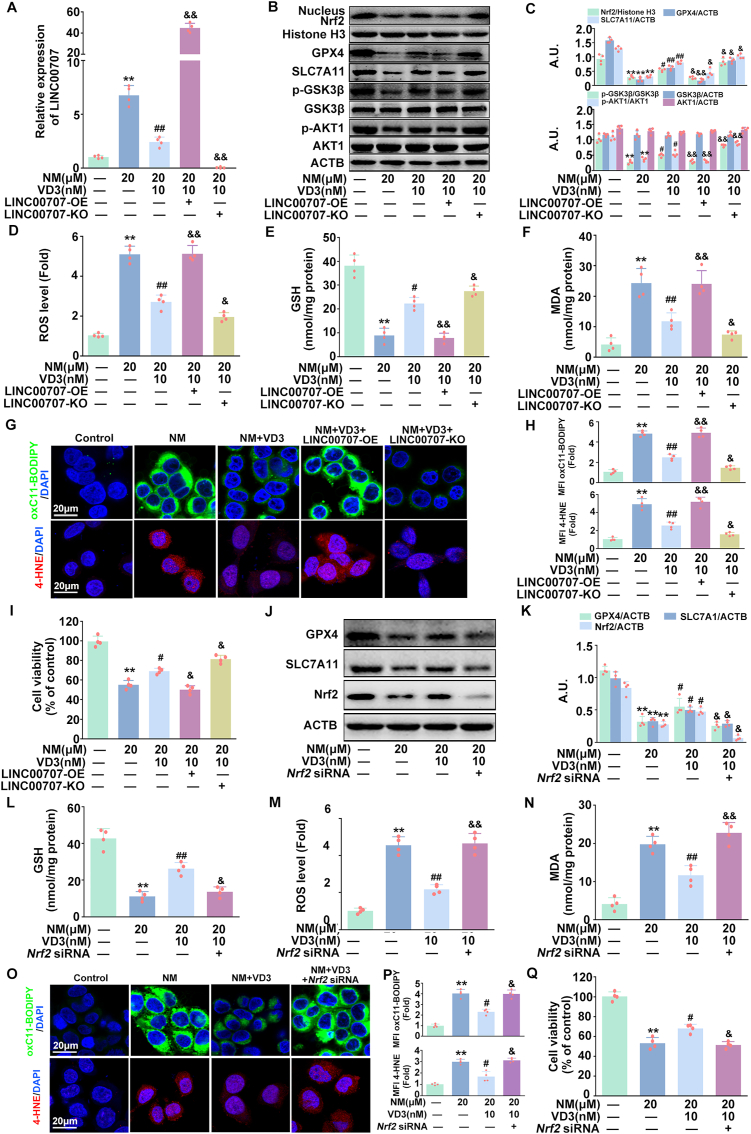
**VD3 inhibited ferroptosis via the LINC00707-AKT1-GSK3β-Nrf2 signaling pathway in keratinocytes.** Cells transfected with or without LINC00707 overexpression or knockout lentivirus were pretreated with VD3 (10 nM) for 1 h, followed by treatment with NM (20 μM) for another 24 h. **(A)** The expression of LINC00707 was detected by qRT-PCR. **(B)** The proteins levels of nuclear Nrf2, Histone H3, GPX4, SLC7A11, *p*-GSK3β, GSK3β, *p*-AKT1, AKT and ACTB were detected by Western blot. **(C)** The bar graphs show the quantification of the indicated proteins. **(D)** Detection of ROS levels using DCFH-DA. **(E)** Assay of GSH content with a specific kit following the manufacturer's instructions. **(F)** MDA levels were detected by a lipid peroxidation assay kit. **(G)** Representative images of oxC11-BODIPY or 4-HNE detected with a ZEISS LSM800 confocal laser scanning microscope. **(H)** Relative MFI of oxC11-BODIPY or 4-HNE. **(I)** Cell viability was detected by a CCK-8 detection kit. Cells were transfected with *Nrf2* siRNA as described in Materials and methods. At 24 h post-transfection, cells were pretreated with VD3 (10 nM) for 1 h, followed by the incubation of NM (20 μM) for an additional 24 h. **(J)** Western blot analysis of GPX4, SLC7A11, Nrf2 and ACTB proteins levels. **(K)** The bar graphs show the quantification of the indicated proteins. **(L)** Detection of ROS levels using DCFH-DA. **(M)** Assay of GSH content with a specific kit following the manufacturer's instructions. **(N)** MDA levels were detected by a lipid peroxidation assay kit. **(O)** Representative images of oxC11-BODIPY or 4-HNE detected witha ZEISS LSM800 confocal laser scanning microscope. **(P)** Relative MFI of oxC11-BODIPY or 4-HNE. **(Q)** Cell viability was detected by a CCK-8 detection kit. Values are expressed as means ± SD (n = 4); ∗*P* < 0.05, ∗∗*P* < 0.01 vs. the vehicle-treated control group; ^#^*P* < 0.05, ^##^*P* < 0.01 vs. NM-treated group. ^&^*P* < 0.05, ^&&^*P* < 0.01 vs. NM and VD3 co-treated group.

Our above findings have confirmed that LINC00707 plays a core role in NM-induced ferroptosis, therefore, the potential relationship between LINC00707 and VD3 in NM-treated keratinocytes was explored. As shown in Fig. 7A–C, VD3 decreased LINC00707 expression, increased *p*-AKT1, *p*-GSK3β, nuclear Nrf2, GPX4 and SLC7A11 expression in NM-stimulated keratinocytes. LINC00707-OE transfection reversed the effect of VD3 on the expression of LINC00707, *p*-AKT1, *p*-GSK3β, nuclear Nrf2, GPX4, and SLC7A11, the production of ROS, GSH, MDA, lipid ROS and 4-HNE and cell viability in NM-treated keratinocytes, which was enhanced by LINC00707-KO transfection (Fig. 7A–I). Our results highlighted the involvement of LINC00707-AKT1-GSK3β pathway in the protective effect of VD3 against NM-induced ferroptosis in keratinocytes.

Moreover, *Nrf2* siRNA markedly abolished VD3-induced increase of GPX4, SLC7A11 expression and 10.13039/501100022272GSH production, and the inhibition of ROS, MDA, lipid ROS and 4-HNE generation and cell death in NM-treated keratinocytes (Fig. 7J-Q), supporting that Nrf2 was required in VD3-induced ferroptosis inhibition. Accordingly, we concluded that the LINC00707-AKT1-GSK3β-Nrf2 pathway was critical for VD3-induced inhibition of ferroptosis in NM-exposed keratinocytes.

### 3.7 VD3 attenuated NM-induced dermal toxicity through the inhibition of ferroptosis via the LINC00707-AKT1-GSK3β-Nrf2 signaling pathway in vivo

**Fig. 8:**
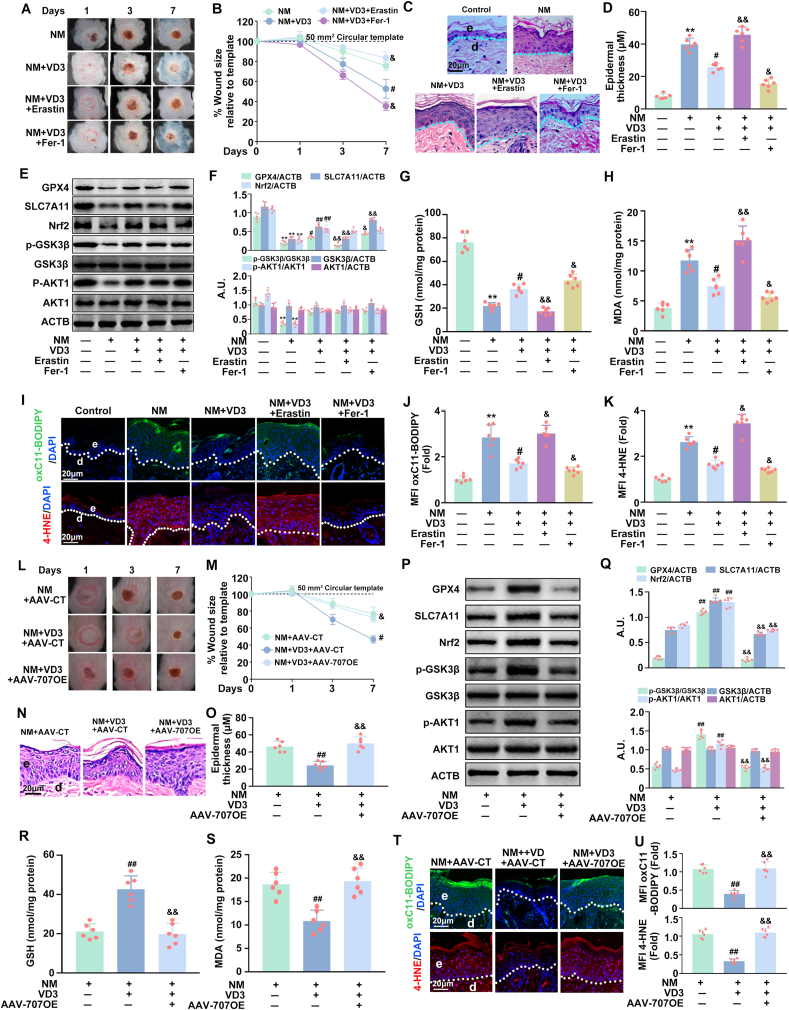
**VD3 attenuated NM-induced dermal toxicity through the inhibition of ferroptosis via the LINC00707-AKT1-GSK3β-Nrf2 signaling pathway *in vivo*.** Dorsal skin of 8 week-old C57BL/6J mice (n = 6 per group) were exposed to NM in the presence or absence of VD3 (50 ng/100 μL per mouse, i.p.) combined with or without erastin (50 mg/kg) or topical application of Fer-1 (10 μM, 200 μL total volume) as described in Materials and method. **(A)** Representative images of skin wounds healing post-NM exposure. **(B)** The quantification of the wound size related to (A). Mice were sacrificed at 7 d following NM exposure, and dorsal skin tissues were collected. **(C)** H&E staining was performed for analysis of epidermal thickness. **(D)** The quantification of the epidermal thickness related to (C). **(E)** Western blot was used to detect the levels of Nrf2, *p*-GSK3β, GSK3β, *p*-AKT1, AKT1, GPX4, SLC7A11 and ACTB proteins expression. **(F)** The bar graphs show the quantification of the indicated proteins. **(G)** Assay of GSH content with a specific kit following the manufacturer's instructions. **(H)** MDA levels were detected by a lipid peroxidation assay kit. **(I)** Representative fluorescence images of mice dorsal skin wounds and nearby tissues stained with either C11-BODIPY or 4-HNE, then visualized by a ZEISS LSM800 confocal laser scanning microscope. Relative MFI of **(J)** C11-BODIPY or **(K)** 4-HNE. Mice were subcutaneously injected with 10 μL of AAV-707OE (1.8 × 10^13^ vg) or AAV-CT (1.8 × 10^13^ vg) as described in Materials and method. **(L)** Representative images of skin wounds healing post-NM exposure. **(M)** The quantification of the wound size related to (L). Mice were sacrificed at 7 d following NM exposure, and dorsal skin tissues were collected. **(N)** H&E staining was performed for analysis of epidermal thickness. **(O)** The quantification of the epidermal thickness related to (N). **(P)** Western blot was used to detect the levels of Nrf2, *p*-GSK3β, GSK3β, *p*-AKT1, AKT1, GPX4, SLC7A11 and ACTB proteins expression. **(Q)** The bar graphs show the quantification of the indicated proteins. **(R)** Assay of GSH content with a specific kit following the manufacturer's instructions. **(S)** MDA levels were detected by a lipid peroxidation assay kit. **(T)** Representative fluorescence images of mice dorsal skin wounds and nearby tissues stained with either C11-BODIPY or 4-HNE, then visualized by a ZEISS LSM800 confocal laser scanning microscope. **(U)** Relative MFI of C11-BODIPY or 4-HNE. Values are expressed as means ± SD (n = 6); ∗*P* < 0.05, ∗∗*P* < 0.01 vs. the vehicle-treated control group; ^#^*P* < 0.05, ^##^*P* < 0.01 vs. NM-treated group. ^&^*P* < 0.05, ^&&^*P* < 0.01 vs. NM and VD3 co-treated group. e, epidermis; d, dermis; A.U., arbitrary units.

Finally, we ascertained whether VD3-mediated suppression of NM-induced skin injury involves a similar mechanism in vivo. As shown in Fig. S1, NM had no significant effect on serum 25(OH)D; a single dose of 50 ng VD3 (cholecalciferol) significantly increased the serum concentration of 25 (OH)D (a robust and reliable marker of vitamin D status) [41], which could last for at least 7 days. VD3 accelerated wound healing and promoted maturation of new epidermis, as evident from examination of the epidermal thickness of NM-exposed skin (Fig. 8A–D). Meanwhile, VD3 induced a significant upregulation of *p*-AKT1, *p*-GSK3β and nuclear Nrf2 expression, thereby inhibiting ferroptosis in details with increased GPX4, SLC7A11 expression and GSH contents, and decreased MDA, lipid ROS and 4-HNE generation (Fig. 8E–K). Moreover, erastin notably abolished the beneficial effects of VD3 on NM-caused dermal toxicity, GPX4 and SLC7A11 expression, GSH depletion, MDA production, lipid ROS and 4-HNE generation, which was enhanced by Fer-1 (Fig. 8A–K). Finally, the protective effects of VD3 against NM-caused dermal toxicity, *p*-AKT1, *p*-GSK3β and nuclear Nrf2 expression, and ferroptosis were markedly eliminated by AAV-707OE in mice (Fig. 8L-U). Hence, the present data implied the involvement of the LINC00707-AKT1-GSK3β-Nrf2 signaling pathway in VD3-mediated suppression of ferroptosis and the subsequent mitigation of NM-induced dermal toxicity in vivo.

## 4 Discussion

NM triggers severe skin damage characterized by delayed blister formation, with a lack of effective and targeted therapies. A significant constraint lies in the incomplete comprehension of the precise mechanism underlying NM-induced injury. In the present study, we identified for the first time that NM-caused dermal toxicity through the induction of ferroptosis.

Ferroptosis is a newly defined type of programmed cell death that is mediated by ROS accumulation and iron-dependent lipid peroxidation. Emerging studies have shown that ferroptosis plays an essential role in the pathophysiological process of multiple diseases including skin disorders [42]. Compared with normal lesions, lipid ROS and ferrous iron, the expression of acyl-CoA synthetase long-chain family member 4 (ACSL4) were increased, and the expression of GPX4 was decreased in psoriatic lesions, Fer-1 significantly suppresses ferroptosis and alleviates psoriasiform dermatitis [6,7]. Cui et al. demonstrated that secretory autophagosome derived from human umbilical vein endothelial cells inhibits free Fe^2+^-mediated ferroptosis, thereby restoring the functions of skin repair cells, ultimately accelerating diabetic wound healing [43]. Another study from Du and his colleagues pointed out that electroacupuncture promotes skin wound repair by improving lipid metabolism and inhibiting ferroptosis [44]. Meanwhile, UVB exposure causes excessive ROS accumulation and lipid preoxidation products by elevating ACSL4 expression and reducing GPX4 expression, leading to keratinocyte ferroptosis, which subsequently initiating skin inflammation [8]. NMN inhibits ferroptosis by recruiting GSH to enhance GPX4-mediated ferroptosis defense in keratinocytes, thereby attenuating UV irradiation-stimulated skin injury [9]. These published data unequivocally underscores the necessity of ferroptosis in the pathogenesis of skin disorders. Our present findings validated that NM induced ferroptosis that contributed to NM-caused cytotoxicity in keratinocytes. This work furnishes fresh evidence that ferroptosis contributes substantially to NM-mediated dermal toxicity, implying that directing therapeutic efforts towards ferroptosis could hold promise for treating skin injuries induced by NM.

Furthermore, the underlying mechanisms responsible for NM-triggered ferroptosis were further investigated. Ferroptosis is critically coordinated by the action of several components involved in the metabolism of iron, ROS, and GSH and is regulated by intricate networks such as Nrf2 [13]. Nrf2, a stress-responsive transcription factor, functions as a pivotal regulator of ferroptosis via targeting numerous genes for preventing lipid peroxidation [13]. It has been found that Nrf2 activation significantly inhibits ferroptosis in melanoma, while Nrf2 inactivation induces ferroptosis thereby sensitizing melanoma to immunotherapy; inhibiting Nrf2-mediated ferroptosis notably improves skin wound healing [45]. Herein, we confirmed that Nrf2 also played an important role in NM-induced ferroptosis and dermal toxicity in vitro and in vivo. Moreover, previous studies have indicated that GSK3β is a key negative regulator of Nrf2 activity. GSK3β is a serine/threonine protein kinase that is active in the resting state and continuously regulates cell biological functions by phosphorylating downstream targets. Up to now, AKT is the most well-established upstream kinase for the regulation of GSK3β activity by phosphorylating its Ser-9 residue [46]. Researches have reported that activation of the AKT-GSK3β pathway leads to increase Nrf2 nuclear translocation, which subsequently alleviating Parkinson's disease [47], type 2 diabetes [7,48]and radiation-induced impairment of wound healing [49]. Recently, Liu et al. confirmed that AKT activation suppresses the activity of GSK3β and stabilizes Nrf2, which decreasing sensitivity to ferroptosis in the isocitrate dehydrogenase gene-mutated cancer cells [50]. Another study from Zeng and his colleagues reported that acetaminophen causes liver injury by inducing ferroptosis through the AKT-GSK3β-Nrf2 pathway in mice [51]. Pretreatment with poxadustat attenuates folic acid-induced kidney injury through antiferroptosis via AKT/GSK3β mediated Nrf2 activation [15]. In the present study, we found that NM induced ferroptosis through inhibiting the AKT1-GSK3β-Nrf2 signaling pathway in keratinocytes. Despite the established involvement of the AKT-GSK3β-Nrf2 pathway in regulating ferroptosis across diverse cell types, our findings represented the first demonstration of its crucial role in NM-induced ferroptosis specifically in keratinocytes. This work provides new insight into the mechanism of ferroptosis induced by NM.

In addition to Nrf2, our findings underscore the significant contribution of LINC00707 in NM-induced ferroptosis in keratinocytes. The LncRNA LINC00707 is located on chromosome 10p14 and is involved in a series of biological functions including cell proliferation, apoptosis and inflammation [40]. Dian et al. showed that silencing of LINC00707 reverses IL1β-induced apoptosis and extracellular matrix degradation of osteoarthritis chondrocytes [52]. Likewise, knockdown of LINC00707 obviously alleviates lipopolysaccharide-induced inflammation and apoptosis in MRC-5 cells [53] and PC12 cells [54]. Recently, LINC00707 expression has been found to be distinctly elevated in melanoma, potentially influencing prognosis through the modulation of mitogen-activated protein kinase and various immune and inflammation-associated pathways [55]. These discoveries unanimously indicate that LINC00707 holds great potential as a therapeutic target for numerous diseases including skin disorders. Our current study demonstrated that LINC00707 would directly bind to AKT1 in the cytoplasm, leading to the downregulation of *p*-AKT1 with no effects on the expression of AKT1 mRNA and protein, suggesting that LINC00707 regulated AKT1 activity in a posttranslational modification manner. Moreover, we also clarified that NM induced the expression of LINC00707 that further promoted ferroptosis through the decrease of *p*-AKT1, *p*-GSK3β and nuclear Nrf2 expression in keratinocytes. Meanwhile, LINC00707 knockdown significantly reversed NM-induced inhibition of the AKT1-GSK3β-Nrf2 signaling pathway, thereby reducing ferroptosis, ultimately attenuating NM-caused cutaneous damage. These effects of NM were enhanced by LINC00707 overexpression. Together, the data intensively indicated that LINC00707 was involved in NM-induced ferroptosis through the regulation of the AKT1-GSK3β-Nrf2 signaling pathway in keratinocytes, which may provide new insights into therapeutic strategy to ameliorate NM-induced dermal damage. LncRNAs have been found to play a pivotal role in various skin disorders especially UV-induced skin injury [[20], [21], [22],^56^,^57^]. Our findings are crucial complements to the previous works, suggesting that LncRNAs would be good candidates of targets for skin wound healing.

Ultimately, our results underscore the crucial role of the LINC00707-AKT1-GSK3β-Nrf2 signaling pathway and ferroptosis in mediating the protective effect of VD3 against NM-elicited cutaneous toxicity. VD3 is a widely available fat-soluble secosteroid for maintaining health as a necessary nutrient in human. It has been found to be not only produced and metabolized in skin, but also regulate epidermal proliferation and differentiation thereby promoting wound healing [58]. Considerable evidence supports that VD3 is a key protector for NM-caused cutaneous injuries by alleviating inflammation [23] and preventing cell death and tissue destruction [7,34], however, the precise mechanisms require additional clarification. Our current findings validated that VD3 significantly mitigated NM-induced skin injury and ferroptosis in vitro and in vivo, which was abolished by erastin and enhanced by Fer-1. Meanwhile, VD3 attenuated NM-caused decrease of Nrf2 nuclear translocation. *Nrf2* siRNA transfection notably reversed VD3-induced inactivation of ferroptosis. Moreover, VD3 also suppressed NM-mediated expression of LINC00707, inhibition of *p*-AKT1, *p*-GSK3β and nuclear Nrf2 expression, which was further enhanced by knockdown of LINC00707 and was blocked by LINC00707 overexpression in keratinocytes. Although the exact mechanisms by which VD3 inhibited LINC00707 expression remain to be elucidated, our present data indicated that VD3 is a feasible treatment option for NM-induced dermal toxicity by inactivating ferroptosis, which was partially mediated through the LINC00707-AKT1-GSK3β-Nrf2 signaling pathway. Previous researches reported that LncRNAs expression profiles are significantly rearranged by VD3 via vitamin D receptor (VDR) in multiple tumors [59]. Zhu et al. found that VDR activated by VD3 could inhibit the progression of colorectal cancer through directly binding to the LncRNA maternally expressed gene 3 promoter and regulating its expression [60]. More recently, Jiang et al. showed that VDR depletion markedly increased the expression of H19, HOTTIP and Nespas, whereas decreasing the expression of Kcnq1ot1, lincRNA-p21 in keratinocytes [61]. Whether VD3 mitigates NM-induced upregulation of LINC00707 via the VDR signaling in keratinocytes needs further studies.

## 5 Conclusion

**Fig. 9:**
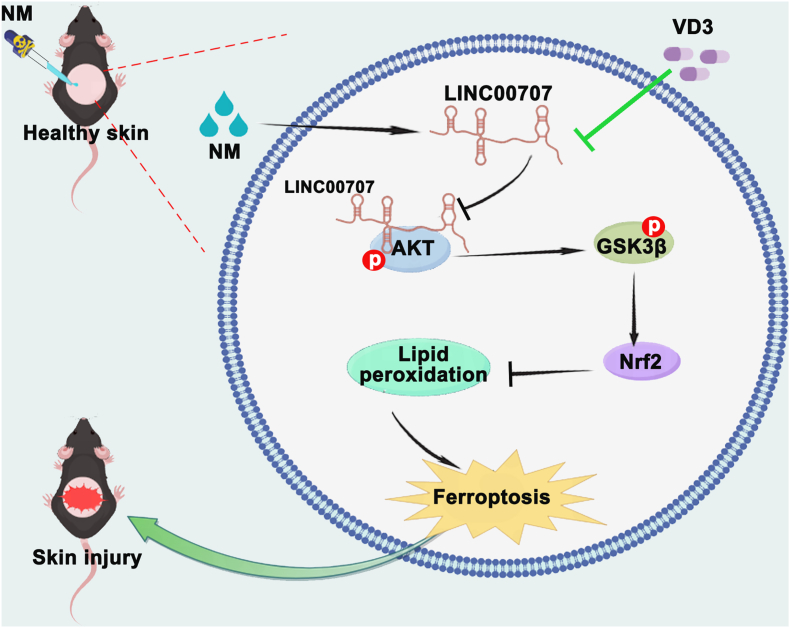
VD3 attenuated NM-caused dermal toxicity by inhibiting ferroptosis through the LINC00707-AKT1-GSK-3β-NRF2 signaling pathway.

In conclusion, we elucidated a functional link between LINC00707 and Nrf2-dependent ferroptosis in NM-caused cutaneous injury. NM notably increased LINC00707 expression which could directly bound with AKT1 to block its phosphorylation, thereby activating GSK3β-mediated Nrf2 inactivation, subsequently inducing ferroptosis. Interestingly, VD3 supplementation mitigated NM-caused dermal toxicity by inhibiting ferroptosis through decreasing the expression of LINC00707 and then activating the AKT1-GSK3β-Nrf2 signaling pathway (Fig. 9). Our collective discoveries pave a novel avenue for investigating the underlying mechanisms of VD3's protective effect against NM-induced dermal toxicity, indicating that targeting LncRNAs or inhibiting ferroptosis would emerge as potent therapeutic approaches to combat vesicants-induced skin wound healing.

## CRediT authorship contribution statement

**Xunhu Dong:** Writing – original draft, Conceptualization, Data curation, Formal analysis, Investigation, Methodology, Resources, Software, Validation, Visualization. **Ying He:** Writing – original draft, Conceptualization, Data curation, Formal analysis, Investigation, Methodology, Resources. **Xiaofeng Hu:** Data curation, Formal analysis, Investigation, Methodology, Resources. **Jie Wu:** Data curation, Formal analysis, Investigation, Methodology, Resources. **Feng Ye:** Data curation, Formal analysis. **Xiaogang Wang:** Data curation, Methodology, Formal analysis. **Yuanpeng Zhao:** Data curation, Methodology, Formal analysis. **Guorong Dan:** Resources, Data curation, Formal analysis. **Jiqing Zhao:** Data curation, Formal analysis. **He Tang:** Data curation, Formal analysis. **Xiaolu Lu:** Investigation, Data curation. **Yan Sai:** Writing – review & editing, Funding acquisition, Project administration, Resources, Supervision, Validation, Visualization. **Zhongmin Zou:** Writing – review & editing, Conceptualization, Funding acquisition, Project administration, Supervision, Validation, Visualization. **Mingliang Chen:** Writing – review & editing, Conceptualization, Data curation, Formal analysis, Funding acquisition, Investigation, Methodology, Project administration, Resources, Software, Supervision, Validation, Visualization, Writing – original draft.

## Data availability

All data and materials are available to the researchers once published.

## Funding sources

This work was supported by the 10.13039/501100001809National Natural Science Foundation of China (grant number: 82202189 and 82073544), 10.13039/501100007957Chongqing Municipal Education Commission Science and Technology Research Project (grant number: KJZD-K202412804), Chongqing Youth Innovation Talent Project (grant number: CSTB2024NSCQ-QCXMX0036), Science and Technology Innovation Key R&D Program of Chongqing (grant number: CSTB2024TIAD-STX0045), Special postdoctoral Foundation of Chongqing (grant number: 2023CQBSHTBT002), 10.13039/501100002858China Postdoctoral Science Foundation (grant number: 2023TQ0148), Talent Incubation Program for Young Doctors at the Second Affiliated Hospital of 10.13039/501100012397Army Medical University (grant number: 2023YQB032).

## Declaration of competing interest

The authors declare that they have no known competing financial interests or personal relationships that could have appeared to influence the work reported in this paper.
