# Supplementary material for: Targeting LINC00707 by vitamin D3 attenuates nitrogen mustard-caused dermal toxicity through inhibiting ferroptosis
Source: Redox Biol. 2025 Apr 10;83:103628. doi: 10.1016/j.redox.2025.103628 (PMC12146658; doi:10.1016/j.redox.2025.103628)
Supplement: Multimedia component 1 [file mmc1.docx]

**Supplementary Materials for:**

**Vitamin D3** **attenuates nitrogen mustard-caused dermal toxicity through inhibiting ferroptosis via the LINC00707-AKT1-GSK3β-Nrf2 signaling pathway**

Xunhu Dong^a,1^, Ying He^b,c,1^, Xiaongfeng Hu^d,1^, Jie Wu^e,1^, Feng Ye ^a^, Xiaogang Wang ^a^, Yuanpeng Zhao ^a^, Guorong Dan ^a^, Jiqing Zhao ^a^, He Tang ^a^, Xiaolu Lu ^b^, Yan Sai ^a, f,^ _*_, Zhongmin Zou ^a,^_**_, Mingliang Chen ^a, f,^ _***_

^a^Institute of Toxicology, School of Military Preventive Medicine, Army Medical University, Chongqing 400038, China.

^b^Institute of Pathology and Southwest Cancer Centre, Southwest Hospital, Army Medical University, Chongqing, 400038, China.

^c^Department of Ultrasound, Xinqiao Hospital, Army Medical University, Chongqing 400037, China.

^d^Chinese PLA Center for Disease Control and Prevention, Beijing, China.

^e^Department of Tropical Medicine, School of Military Preventive Medicine, Army Medical University, Chongqing 400038, China.

^f^State Key Laboratory of Trauma and Chemical Poisoning

_*_Corresponding author. Institute of Toxicology, School of Military Preventive Medicine, Army Medical University, 30 Gaotanyan Street, Shapingba District, Chongqing 400038, China.

_**_Corresponding author. Institute of Toxicology, School of Military Preventive Medicine, Army Medical University, Chongqing 400038, China. E-mail: zmzou@tmmu.edu.cn.

_***_Corresponding author. Institute of Toxicology, School of Military Preventive Medicine, Army Medical University, 30 Gaotanyan Street, Shapingba District, Chongqing 400038, China. E-mail: chenml@tmmu.edu.cn

*E-mail addresses:* sai2000cn@tmmu.edu.cn (Y. Sai), zmzou@tmmu.edu.cn (Z. Zou), chenml@tmmu.edu.cn (M. Chen).

^1^ These authors contribute equally to this work.

1. **Materials and Methods**

**1.1 Reagents and antibodies**

RPMI 1640 Medium (SH30809.01B) and fetal bovine serum ([SH30370.03](http://www.bioon.com.cn/reagent/show_product.asp?id=1237790)) were purchased from Hyclone Laboratories (South Logan, UT, USA). Mechlorethamine hydrochloride (a type of NM, K900001X) was obtained from Dibo (Shanghai, China). Erastin (HY-15763), Fer-1 (HY-100579), SC79 (HY-18749), A-674563 (HY-13254), AR (HY-10512), tBHQ (HY-100489), ML385 (HY-100523) and VD3 (HY-10002/HY-15398) were purchased from Med Chem Express (New Jersey, USA), and CCK-8 (CK04) was from Dojindo Laboratories (Kumamoto, Japan). DAPI (C1005), RIPA (P0013B), GSH assay kit (S0053) and DCFH-DA (S0033) was obtained from the Beyotime Institute of Biotechnology (Shanghai, China). Lipofectamine™ 3000 transfection reagent (L3000015), C11-BODIPY (D3861), pierce magnetic RNA-protein pull-down kit (20164) and MEGAscript T7 transcription kit (AMB13345) were acquired from Invitrogen (Carlsbad, CA, USA). DMSO (D2650), Iron assay kit (MAK025), lipid peroxidation assay kit (KAK085), and magna RIP^®^ RNA-binding protein immunoprecipitation kit (177001) were purchased from Sigma-Aldrich (St. Louis, MO, USA). Antibodies were obtained as follows: Anti-4-HNE (ab48506), anti-AKT (ab179463), anti-AKT1 (ab233755), anti-p-AKT1 (S473, ab314038), anti-AKT2 (ab175354), anti-p-AKT2 (S474, ab38513), anti-AKT2 (ab152157), anti-Nrf2 (ab62352), and anti-DDDDK tag (Flag, ab205606) were obtained from Abcam (Cambridge, UK) and anti-p-AKT (Ser473, 4060) was from Cell Signaling Technology (Beverly, MA, USA). anti-p-AKT3 (Ser472, bs-5209R) was purchased from Bioss (Beijing, China). Anti-GSK3β (22104-1-AP), anti-p-GSK3β (Ser9, 67558-1), GPX4 (67763-1) and SLC7A11 (26864-1-AP) were acquired from Proteintech (Wuhan, China). Antibodies against β-actin (ACTB, AF2811) and Histone H3 (AF0009) were purchased from the Beyotime Institute of Biotechnology (Shanghai, China).

**1.2 Cell viability measurements**

The CCK-8 was utilized to assess cell viability, adhering to the manufacturer's outlined protocol as previously described ^[1](#_ENREF_1),[2](#_ENREF_2)^. In brief, HaCaT cells were seeded into a 96-well microplate (Corning Life Science; 3650) at a density of 5,000 cells per well. After 24 h, the medium was replaced, and the cells were exposed to various concentrations of NM (1, 5, 10, 20, and 50 μM) for 24 h. Following this treatment, 20 μL of CCK-8 solution was added to each well and incubated for 1.5 h. Subsequently, the number of viable cells was quantified by measuring absorbance at 450 nm using a monochromator microplate reader (Safire II; Tecan Group Ltd., Männedorf, Switzerland). The optical density reading at 450 nm was then converted into a percentage of cell viability, with the control group serving as the baseline (set at 100%).

**1.3** **Detection of intracellular ferrous iron (Fe^2+^) and total iron** **content**

The intracellular Fe^2+^ and total iron concentrations were ascertained using a designated iron assay kit, adhering to the manufacturer's guidelines. Specifically, 2 × 10^6^ HaCaT cells were collected and homogenized in 4 to 10 volumes of iron assay buffer, followed by centrifugation at 16,000 × g for 10 minutes at 4°C to eliminate insoluble components. Subsequently, the samples were diluted with assay buffer to a final volume of 100 μL and incubated with either 5 μL of iron assay buffer or iron reducer in 96-well plates for 30 minutes at 25°C in the dark, to detect intracellular Fe^2+^ or total iron levels, respectively. An iron probe was then added to each well and incubated for an additional 60 minutes at 25°C. Finally, the concentrations of Fe^2+^ and total iron were quantified using an Infinite^TM^ M200 Microplate Reader (Tecan Group Ltd.) at a wavelength of 593 nm.

**1.4 Measurement of GSH and MDA contents**

Following the application of various treatments, cells or skin tissues were harvested and lysed with RIPA lysis buffer. Subsequently, GSH levels were quantified using the GSH Assay kit, and MDA levels were determined through the Lipid peroxidation assay kit, both according to the manufacturer's instructions.

**1.5 ROS assessments**

Levels of ROS were quantified utilizing DCFH-DA in accordance with the manufacturer's guidelines as previously detailed^[2](#_ENREF_2)^. HaCaT cells were plated onto a 96-well microplate at a density of 5,000 cells per well and subjected to the specified treatments. Subsequently, the cells were incubated with DCFH-DA (10 μM) at 37°C for 30 minutes under dark conditions, followed by three gentle rinses with warm phosphate-buffered saline. The fluorescence intensity corresponding to total ROS was then measured using an InfiniteTM M200 Microplate Reader (Tecan Group Ltd.).

**1.6 Quantitative reverse transcriptase polymerase chain reaction (qRT-PCR) analysis**

A Trizol reagent (Invitrogen, USA) were applied to extract the total RNAs from cells. The total RNA using a PrimeScript^TM^ RT reagent kit (Takara, Japan) was used to generate cDNAs, followed by qRT-PCR using a SYBR^®^ Premix Ex TaqTM II reagent (Takara) according to the manufacturer’s protocols. All ampliﬁcation reactions were performed in triplicate, and the averages of the threshold cycle (Ct) were used to interpolate curves using 7300 System SDS Software. *ACTB* served as internal reference genes. The sequence of the primers were shown in Supplementary **Table. S1**.

**1.7 Western blot analysis**

Cells or skin tissues were collected, lysed or homogenized for protein extraction and subjected to western blot, as described previously^[2](#_ENREF_2)^. Briefly, 40-60 µg protein was resolved via 12-15% SDS-PAGE and electroblotted onto polyvinylidene difluoride membranes. Next, membranes were blocked with 5% skimmed milk and incubated overnight at 4℃ with primary antibodies at the following dilutions: AKT (1:1000), p-AKT (1:1000), AKT1 (1:1000), p-AKT1 (1:1000), AKT2 (1:1000), p-AKT2 (1:500), AKT3(1:1000), p-AKT3 (1:1000), GSK-3β (1:500), p-GSK-3β (1:1000), Nrf2 (1:1000), GPX4 (1:1000), SLC7A11 (1:500), Histone H3 (1:1000) and ACTB (1:1000). After washing three times with Tris-buffered saline containing 0.1% Tween 20 (TBST), membranes were incubated with the appropriate horseradish peroxidase-conjugated secondary antibodies ([Thermo Scientific Lab Vision;](http://www.thermoscientific.com/ecomm/servlet/search?searchType=0&searchSubType=6&N=4294967135%204294967089&Ne=4294967089&keyWord=rabbit+secondary+antibodies) 31340 and 31455). Following TBST washes, protein bands were visualized with electrogenerated chemiluminescence using the Vilber Fusion FX7 system.

**1.8** **Fluorescence microscopy**

HaCaT cells were plated in 15-mm confocal dishes and treated as indicated. Then, cells were stained with C11-BODIPY 581/591 sensor according to the manufacturer’s protocol, fixed with 4% paraformaldehyde, permeabilized with 0.3% Triton X-100 in PBS for 15 min, blocked with 3% BSA in phosphate-buffered saline (PBS) for 45 min and washed with 0.5% BSA in PBS. At 7 d after NM exposure, the wounded skin and nearby control tissues were excised in full-thickness and embedded in OCT at -20°C. Then, the tissues were sectioned at a thickness of 6-8 μm. Fresh mice dorsal skin tissue were treated with C11-BODIPY 581/591 sensor prior to fixation. Meanwhile, for immunofluorescence analysis, the cells and sections were washed three times with PBS for 5 min, permeabilized with 0.3% (v/v) Triton X-100 for 10 min and blocked in 3% BSA for 1 h at room temperature. Thereafter, the cells and sections were incubated with mouse anti-4-HNE antibody (1:200) overnight at 4°C. Then, the cells and sections were incubated with the appropriate Alexa Fluor® 594 goat anti-mouse IgG (H+L) antibody (A11005, Invitrogen) for 2 h at room temperature. DAPI staining solution was used to staining the nuclei for 10 min at room temperature. Finally, the sections were mounted on glass slide. All the digital images of the dishes and sections were acquired using a ZEISS LSM800 confocal laser scanning microscope (ZEISS, Germany).

**1.9 Transmission electron microscopy**

HaCaT cells were collected and fixed in 2% paraformaldehyde and 0.1% glutaraldehyde in 0.1 M sodium cacodylate for 2 h, post-fixed with 1% OsO4 for 1.5 h, washed, and stained for 1 h in 3% aqueous uranyl acetate. The samples were then washed again, dehydrated with graded alcohol, and embedded in Epon-Araldite resin (Canemco & Marivac, 034). Ultrathin sections were cut on a ultramicrotome (Reichert-Jung, Inc., Cambridge, UK), counterstained with 0.3% lead citrate, and examined on a transmission electron microscope (model no.: EM420; Koninklijke Philips Electronics N.V., Amsterdam, The Netherlands).

**1.10 LncRNA expression profiling array**

Total RNA was extracted utilizing Trizol reagent sourced from Takara (Dalian, China), with the quantity and quality of the RNA confirmed through standard denaturing agarose gel electrophoresis and NanoDrop 2000 spectrophotometry (Thermo, USA). The expression profiles of long non-coding RNAs (lncRNAs) were examined using the Arraystar Human LncRNA Microarray V3.0, a comprehensive platform designed for global profiling of human lncRNAs and protein-coding transcripts. This microarray encompasses 30,586 lncRNA probes and 26,109 protein-coding transcript probes. RNA labeling and array hybridization were carried out according to the Agilent One-Color Microarray-Based Gene Expression Analysis protocol (Agilent Technology, USA). In summary, the target RNAs were isolated from total RNA by eliminating rRNA with an mRNA-ONLYTM Eukaryotic mRNA Isolation kit (Epicentre, USA). Fluorescently labeled cDNAs were synthesized with random primers, purified with a RNeasy Mini Kit (Qiagen, Germany), and subsequently hybridized to the Human LncRNA Microarray (Arraystar). Following hybridization and washing, the slides were scanned with the Agilent DNA Microarray Scanner (G2505C model). The resulting data were analyzed using GeneSpring GX v12.1 software (Agilent Technologies).

**1.11 Lentivirus and plasmids construction**

The lentiviral vectors encoding LINC00707 DNAs (including sense, antisense and truncations), AKT1 (WT and three sets of domain truncation mutants) CDS sequence flanking with flag DNA, Nrf2 and lentiviral vetors carrying a LINC00707 short hairpin RNA were designed and synthesized by Gene Chem (Shanghai, China). The processes of transduction and the establishment of stable cell lines were executed according to the manufacturer’s instructions.

**1.12 siRNA assay**

HaCaT cells were transfected with *AKT1* siRNA (sc-35527, Santa cruz biotechnology) or negative control siRNA (sc-44230, Santa cruz biotechnology) for 24 h, in keeping with the manufacturer’s protocol. Cells were subsequently washed and incubated with fresh RPMI-1640 for a further 24 h. Next, cells were harvested for evaluation of target protein expression or incubated with specific reagents. At the end of the incubation period, cells were harvested and subjected to western blot and other analyses.

**1.13 Fluorescence in situ hybridization and immunofluorescence staining**

The detection of LINC00707 in situ was performed utilizing a Fluorescent In Situ Hybridization Kit (C10910, sourced from RIBOBIO, Guangzhou, China), adhering strictly to the manufacturer's specified protocol. For colocalization of LINC00707 with AKT1, the cells were further incubated with mouse anti-AKT1 antibody (diluted 1:250) for an additional 16 h at 4°C after being incubated with the RNA probes overnight at 40 °C in darkness. Following that, the cells underwent a 2-hour incubation process at room temperature with the suitable Alexa Fluor® 594 goat anti-mouse IgG (H+L) antibody (A11005, Invitrogen). The nuclei were stained for 10 minutes at room temperature utilizing DAPI staining solution. Subsequently, all digital images were captured utilizing a ZEISS LSM800 confocal laser scanning microscope (ZEISS, Germany).

**1.14 RIP assay**

Using the RIP® RNA-binding protein immunoprecipitation kit, RIP assays were conducted in adherence to the manufacturer's specified protocol. In summary, the process involved collecting either 1 × 10^7^ HaCaT cells or flag-tagged AKT1-overexpressing cells, subjecting them to centrifugation at 14,000 × g, and storing at 4 °C for further use. A total of 3 μg of either anti-AKT1 or anti-Flag antibody was incubated with beads for an hour at ambient temperature, followed by an overnight incubation of cell lysate with the beads at 4 °C. Subsequently, the proteins bound to the complex were digested utilizing proteinase K buffer at 55 °C for 30 minutes. The RNAs enriched by either anti-AKT1 or anti-Flag antibodies were then purified and dissolved in 20 μl nuclease-free H_2_O. The enrichment of LINC00707 within these RNAs was quantitatively assessed through qRT-PCR experiments, with its relative abundance determined in comparison to a negative control (IgG). Additionally, *ACTB* mRNA acted as an alternative negative control for LINC00707.

**1.15 RNA pull-down assay**

Sangon Company synthesized DNA sequences that corresponded to either the sense or antisense strands of LINC00707, with each sequence incorporating a T7 promoter sequence at its 5’ end. In vitro transcription was performed to generate LINC00707 using an In Vitro Transcription Kit (AM1334, ThermoFisher) adhering to the manufacturer's guidelines. Subsequently, biotinylated dCTP was attached to the 3’ end of the LINC00707 utilizing the Pierce RNA 3’ End Desthiobiotinylation Kit (20163, ThermoFisher), following the manufacturer's instructions. RNA pull-down assays were then conducted with the PierceTM Magnetic RNA-Protein Pull-Down Kit (20164, ThermoFisher), also adhering to the specified protocol. Lastly, the isolated proteins were identified by western blot. Primer sequences for transcription in vitro were given in Supplementary **Table. S1**.

**1.16 Detection of serum 25(OH)D contents**

Serum 25(OH)D levels of mice were measured by an enzyme-linked immunosorbent assay using a commercial kit (VID21-K01, Eagle Biosciences, New Hampshire, USA), following manufacturer’s instructions.

**1.17 H&E staining and histopathological analysis**

At 7 d after NM exposure, the skin wounds and adjacent tissues were excised and preserved in 4% paraformaldehyde solution supplemented with 0.1% DEPC for histological examination. Subsequent preparation for paraffin embedding was performed based on routine protocols. For histopathological assessment, 5 μm thick sections were obtained and subjected to H&E staining. Microscopic analysis (using Carl Zeiss, Germany) was then conducted to evaluate characteristics such as epidermal thickness, parakeratosis, epidermal desquamation, and epidermal necrosis, as previously^[3](#_ENREF_3)^.


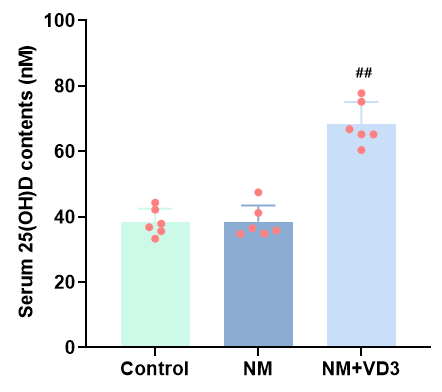


**Figure S1. Serum contents of 25 (OH)D in mice, related to Figure 8.** Serum 25(OH)D levels of mice were measured by an enzyme-linked immunosorbent assay using a commercial kit. Values are expressed as the mean ± SD (*n* = 6); ^##^*p* < 0.01 *vs.* NM-treated group.

**Supplementary Table. S1** The sequence of PCR primer

| Primers | Sequences |
| --- | --- |
| *GPX4* | Forward:5’--3’CCTTTGCCGCCTACTGAA |
|  | Reverse:5’--3’ATGTGCCCGTCGATGTCC |
| *SLC7A11* | Forward:5’--3’TGCCCTTTCCCTCTATTC |
|  | Reverse:5’--3’CGACATTATTCTAAACCACC |
| *AKT1* | Forward:5’--3’CCTCTGCTTTGTCATGGAGTACG |
|  | Reverse:5’--3’AGCCCGAAGTCTGTGATCTTAAT |
| *RP1-124C6.1* | Forward:5’--3’TTGGCCACATGGTGACTCAG |
|  | Reverse:5’--3’TCTGACATTGCGAGCTGGAC |
| *CTA-392E5.1* | Forward:5’--3’CCTCACCAGCCATGTGGAACTAG |
|  | Reverse:5’--3ACTGTTGAAGAAGTCACCAGTAGCG |
| *AC019349.5* | Forward:5’--3’GGCGACCAGAGGCATTAGAG |
|  | Reverse:5’--3’ATAGTGACTGCAGGACCCGT |
| *RP11-54O7.3* | Forward:5’--3’GCTCTGAGGACAGTTGGGAATCTTC |
|  | Reverse:5’--3’GAGGACAGAGGGCTCAGGAAGG |
| *CTD-3099C6.9* | Forward:5’--3’ATCAAGCAATCCATGGTATAGGGAA |
|  | Reverse:5’--3’AGCTTGACTGAAGACCTTGCTG |
| *LINC00941* | Forward:5’--3’GCAGGTCAGGTTATGCAACG |
|  | Reverse:5’--3’GGGTTGGTCTCAGAGGGACT |
| *XLOC_041762* | Forward:5’--3’TGAGCAGACCAGGAAAGGGAGTC |
|  | Reverse:5’--3’GAAGCACAGCATCACAGGGAGAC |
| *XLOC_069528* | Forward:5’--3’TCTTCCACTGGATGTTGCAGG |
|  | Reverse:5’--3’TGCCTTGGTCCATCTTCCAAT |
| *XLOC_009684* | Forward:5’--3’AGCTGGCTGGGCATTAGGTA |
|  | Reverse:5’--3’CCACGAGGCTTCTGCTTACT |
| *LINC00707* | Forward:5’--3’TTGAGAAAGCCACCTCCCAC |
|  | Reverse:5’--3’CACGGTGGCAGTATGGTGAA |
| *ACTB (human)* | Forward:5’--3’CACCCAGCACAATGAAGATCAAG |
|  | Reverse:5’--3’TCATAGTCCGCCTAGAAGCATTT |
| *ACTB (mouse)* | Forward:5’--3’ACTGTCGAGTCGCGTCC |
|  | Reverse:5’--3’CTGACCCATTCCCACCATCA |
| *RIP Primers U1 snRNA* | Forward:5’--3’GGGAGATACCATGATCACGAAGGT |
|  | Reverse:5’--3’CCACAAATTATGCAGTCGAGTTTCCC |
| *LINC00707 probe for FISH* | 5’-cy3-UGGCUUGACUGUGAGUCAUCAUUGCCUAUCU-3’ |

**Reference**

1 Chen, M. L. *et al.* Resveratrol attenuates vascular endothelial inflammation by inducing autophagy through the cAMP signaling pathway. *Autophagy* **9**, 2033-2045, doi:10.4161/auto.26336 (2013).

2 Trimethylamine-N-Oxide Induces Vascular Inflammation by Activating the NLRP3 Inflammasome Through the SIRT3-SOD2-mtROS Signaling Pathway. *J Am Heart Assoc* **6**, doi:10.1161/JAHA.117.002238 (2017).

3 Das, L. M. *et al.* Early indicators of survival following exposure to mustard gas: Protective role of 25(OH)D. *Toxicol Lett* **248**, 9-15, doi:10.1016/j.toxlet.2016.02.013 (2016).
